# Advances in Nanostructured Catalysts for Energy and Environmental Applications

**DOI:** 10.3390/nano16201327

**Published:** 2026-10-20

**Authors:** Jinyou Zheng, Songjie Li

**Affiliations:** State Key Laboratory of Coking Coal Resources Green Exploitation, Engineering Research Center of Advanced Functional Material Manufacturing of Ministry of Education, School of Chemical Engineering, Zhengzhou University, Zhengzhou 450001, China

Escalating global energy demand and the concomitant environmental deterioration, driven by the relentless combustion of fossil fuels, have placed sustainable energy conversion and environmental remediation technologies at the forefront of scientific research. Among the myriad strategies pursued, nanostructured catalysts have emerged as a cornerstone, offering unprecedented opportunities to modulate catalytic performance through precise control over electronic structure, surface atomic arrangement, and morphology [1,2]. A catalyst’s reactivity is intrinsically governed by its electronic and surface atomic structures, which in turn depend strongly on its nanostructural features. Consequently, the rational design and control of catalyst nanostructures represent some of the most effective pathways to enhanced catalytic performance.

This Special Issue, entitled “Advances in Nanostructured Catalysts for Energy and Environmental Applications”, was conceived to promote advances in the synthetic strategies of nanostructured catalysts, encompassing crystal facet engineering, heterostructure construction, band gap engineering, morphology tailoring, plasmonic coupling, and co-catalyst loading, for applications in photo-/electro-/photoelectrochemical water splitting, the reduction of CO_2_ and N_2_ into value-added chemicals, the photodegradation of organic pollutants, and anti-corrosion. This Special Issue brings together high-quality original research articles and a comprehensive review that collectively address pressing challenges in the field.

The five articles published in this Special Issue span a broad spectrum of catalytic processes, including electrocatalytic hydrogen evolution, photoelectrochemical water splitting, photocatalytic and electrochemical CO_2_ reduction, and oxygen reduction reactions. They can be thematically organized into three interconnected areas: (i) catalytic water splitting for hydrogen production, (ii) CO_2_ conversion into value-added chemicals, and (iii) advanced electrocatalysts for oxygen reduction in energy conversion devices.

Hydrogen, as a clean and high-energy-density energy carrier, is widely regarded as a cornerstone of the future energy landscape. Two contributions in this Special Issue address hydrogen production through distinct but complementary catalytic pathways. Jin et al. [3] report the synthesis of nitrogen-doped Co_6_Mo_6_C supported on nitrogen-doped graphene (N-Co_6_Mo_6_C/NC) as a high-performance bimetallic electrocatalyst for the hydrogen evolution reaction (HER) in alkaline media. The catalyst shows an overpotential of 185 mV at 100 mA cm^−2^ and a Tafel slope of 80 mV dec^−1^ in 1.0 M KOH, demonstrating remarkable activity and durability. This work highlights the synergistic benefits of bimetallic carbide nanostructures and N-doped carbon supports, opening a new avenue for designing cost-effective, platinum-free HER electrocatalysts.

In a complementary approach, Jayarathna et al. [4] demonstrate enhanced photoelectrochemical water splitting (PEC-WS) by surface-modulating In_2_S_3_ photoanodes with vertically standing two-dimensional (2D) MoS_2_ nanosheets. The In_2_S_3_/MoS_2_ heterojunction achieves a photocurrent density of 1.28 mA cm^−2^ at 1.23 V vs. RHE, more than 13 times higher than that of pristine In_2_S_3_, and a maximum photoconversion efficiency of 0.75%. The improved performance is attributed to the extended visible-light absorption range and the efficient separation and transfer of photogenerated electron–hole pairs facilitated by the type-II heterojunction. This work underscores the effectiveness of heterostructure engineering in overcoming the charge-recombination limitations of single-component photoanodes.

The catalytic conversion of CO_2_ into valuable chemicals and fuels represents a dual-benefit strategy that simultaneously mitigates greenhouse gas emissions and addresses energy storage challenges. Two articles in this Special Issue explore CO_2_ reduction through photocatalytic and electrochemical pathways. Xu et al. [5] developed a 2D graphene oxide (GO)–diketopyrrolopyrrole (DPP) film photocatalyst for highly selective CO_2_ reduction under visible-light irradiation. The optimized GO–DPP50 film achieves a CO production rate of 32.62 µmol g^−1^ h^−1^ with nearly 100% selectivity in aqueous solution without sacrificial agents, representing 2.77-fold and 3.28-fold enhancements over pristine GO and bare DPP, respectively. Mechanistic investigations reveal a synergistic mechanism in which the 2D GO framework provides a high-surface-area substrate and rapid electron-transport channels to suppress charge recombination, while uniformly dispersed DPP nanoparticles enhance visible-light absorption and CO_2_ chemisorption through Lewis acid–base interactions. This work establishes a paradigm for integrating 2D nanomaterials with organic semiconductors in film-based photocatalysts. Altimari et al. [6] investigate the galvanostatic electrodeposition of zinc on carbon paper and its implications for electrochemical CO_2_ reduction (ERC). Through a systematic analysis of deposition potential transients and physical characterization, the authors reveal that the transition from compact crystalline to amorphous mossy deposits proceeds via the electrodeposition of metallic zinc followed by the formation of oxidized zinc structures. Varying the zinc precursor concentration and current density controls the timing of this transition, enabling synthesis of composite zinc/oxidized zinc electrodes with tunable phase ratios. The optimized composite electrodes achieve a Faradaic efficiency to CO of 82% at −1.8 V vs. Ag/AgCl. This study provides fundamental insights into morphology-controlled electrodeposition and offers practical guidelines for designing zinc-based ERC catalysts.

The oxygen reduction reaction (ORR) is pivotal in metal–air batteries and fuel cells, where sluggish ORR kinetics at the cathode significantly limit overall device efficiency. Chen et al. [7] contribute a comprehensive review entitled “Recent Advances in Improving the Alkaline Oxygen Reduction Performance of Atomically Dispersed Metal–Nitrogen–Carbon Catalysts.” The authors systematically elucidate the ORR mechanism on M–N–C catalyst surfaces and summarize the main strategies used in recent years to enhance both catalytic activity and stability, including coordination environment regulation, defect engineering, heteroatom doping, and structural optimization. The review concludes by outlining critical research directions to accelerate the commercialization of M–N–C catalysts, providing a valuable roadmap for the community.

Collectively, the contributions in this Special Issue address several persistent knowledge gaps in nanostructured catalysis. First, while rational heterostructure design is widely recognized as an effective strategy for enhancing charge separation, precise control of interface quality and band alignment remains challenging. Jayarathna et al. [4] show that conformal growth of vertically standing 2D MoS_2_ nanosheets on In_2_S_3_ via metal–organic chemical vapor deposition can create a well-defined type-II heterojunction, offering a reproducible pathway for interface engineering. Second, developing platinum-group-metal-free electrocatalysts for alkaline HER with both high activity and durability remains an active area of research. The N-Co_6_Mo_6_C/NC catalyst reported by Jin et al. [3] illustrates that bimetallic carbide nanoparticles embedded in N-doped carbon can achieve competitive HER performance, highlighting the potential of carbide-based electrocatalysts as alternatives to precious metals. Third, achieving high product selectivity in photocatalytic CO_2_ reduction, particularly without sacrificial agents, remains a significant challenge. The GO–DPP film catalyst by Xu et al. [5] achieves nearly 100% CO selectivity under visible light in pure water, showing that rationally integrating 2D nanomaterials with organic semiconductors can simultaneously optimize charge dynamics and surface chemistry for selective CO_2_ conversion. Fourth, the relationship between catalyst morphology, structure, and CO_2_ electroreduction performance is often complex and system-dependent. Altimari et al. [6] provide a detailed mechanistic understanding of how the transition from compact to mossy zinc structures affects ERC activity, establishing clear design principles for morphology-controlled electrocatalyst synthesis. Finally, the review by Chen et al. [7] consolidates the rapidly growing literature on atomically dispersed M–N–C catalysts for alkaline ORR, identifies consensus strategies and remaining controversies, and provides a much-needed synthesis to guide future experimental and theoretical investigations.

Looking ahead, several research directions merit intensified effort to advance the field of nanostructured catalysts for energy and environmental applications:

(i) Operando and in situ characterization: Developing advanced operando techniques, including in situ X-ray absorption spectroscopy, ambient-pressure X-ray photoelectron spectroscopy, and differential electrochemical mass spectrometry, will be essential to elucidate catalysts’ dynamic structural evolution under working conditions and identify the true active sites [8].

(ii) Machine learning-guided catalyst design: Integrating computational screening, high-throughput experimentation, and machine learning can accelerate the discovery of novel nanostructured catalysts by navigating the vast compositional and structural space more efficiently than traditional trial-and-error approaches [9].

(iii) Scale-up and device integration: While many nanostructured catalysts perform well at the laboratory scale, translating them into practical devices, including membrane electrode assemblies, flow cells, and large-area photoelectrodes, requires sustained attention to scalability, long-term stability, and cost [10].

(iv) Coupled catalytic systems: Integrating multiple catalytic processes, such as coupling CO_2_ reduction with organic oxidation, or combining photoelectrochemical water splitting with CO_2_ reduction, can improve overall energy efficiency and enable the co-production of valuable chemicals [11].

(v) Single-atom and dual-atom catalysts: Continued exploration of atomically dispersed catalysts, including dual-atom sites and high-entropy single-atom alloys, offers opportunities to achieve unprecedented catalytic selectivity and activity through precise coordination-environment engineering [12].

The five articles published in this Special Issue collectively demonstrate that the rational nanostructuring of catalysts, through heterostructure engineering, bimetallic carbide design, 2D material–organic semiconductor integration, morphology-controlled electrodeposition, and atomically dispersed active site engineering, can significantly enhance catalytic performance across a range of energy and environmental applications. We hope that this collection will stimulate further interest and inspire new creative viewpoints in these rapidly evolving research areas, ultimately advancing interdisciplinary cooperation and accelerating the transition toward a sustainable energy future.
